# Early-onset pharyngeal airway collapse in infants: a retrospective single-center study

**DOI:** 10.1186/s12887-023-04436-w

**Published:** 2023-11-28

**Authors:** Wei Qing, Chen Xun, Nong Guangmin, Li Yan, Jiang Min, Yang Ruimin, Li Chunyan, Zhang Xiaobo, Yi Xiang, Liu Jing

**Affiliations:** 1 Department of Pediatrics, the First Affiliated Hospital of Guangxi Medical University, Nanning, 530021 China; 2 Department of Anesthesiology, the First Affiliated Hospital of Guangxi Medical University, Nanning, 530021 China; 3 Department of Radiology, the First Affiliated Hospital of Guangxi Medical University, Nanning, 530021 China; 4 Department of Otolaryngology/Head and Neck Surgery, the First Affiliated Hospital of Guangxi Medical University, Nanning, 530021 China

**Keywords:** Infant, Pharyngeal airway collapse, Clinical feature, Comorbidity, Prognosis

## Abstract

**Background:**

Early-onset pharyngeal airway collapse (PAC) in infants, which presents with onset within 6-months old is relatively rare. This disease has not been given enough attention in clinic. The aim of this study was to explore the clinical features, endoscopic findings and outcomes of early-onset PAC in infants.

**Methods:**

The children of PAC with onset within 6-months old were included. A retrospective study was conducted.

**Results:**

(1) Total 26 cases were included. The age of onset was neonatal period in 20 cases, 1 to 3-months old in 5 cases, and 4 to 6-months old in 1 case. (2) The main clinical manifestations were noisy breathing (26/26), suprasternal retraction (18/26), snoring (14/26) and hypoxic episode (13/26). (3) Based on the endoscopic findings, collapse at the retropalatal level was most common (24/26). (4) Twelve cases underwent pharyngolaryngeal CT examination, which revealed abnormal findings in 7 cases. (5) Fifteen cases were accompanied with the other airway malformations. (6) In the group with comorbidities of cerebral impairment or craniofacial abnormalities, 1 case was lost to follow up, 4 cases died, and 10 cases survived, in which 9 cases had neurodevelopmental disorders. In the group without comorbidities, 2 cases were lost to follow up, 9 cases survived, in which 1 case had neurodevelopmental disorders. The incidence of poor prognosis including death and neurodevelopmental disorders was significantly higher in the group with comorbidities than that without comorbidities (*P<0.01*). (7) An symptomatic improvement of PAC was found in the majority of the survived cases (18/19) with age.

**Conclusions:**

Early-onset PAC in infants usually exhibits varying degrees of relief with age, whereas the cases with comorbidities had a poor prognosis.

## Background

Pharyngeal airway is a critical component of the upper respiratory tract. Collapse of pharyngeal airway can result in airflow limitation, especially during the inspiratory period. Principally, the internal diameter of pharyngeal airway is determined by an interaction between neural regulation of upper airway muscle activity and structural properties of the pharyngeal airway [1, 2]. In the older children, pharyngeal airway collapse (PAC) is mainly secondary to the enlargement of the lymphoid tissue (tonsils and adenoid) [3]. It usually presents as snoring and can result in obstructive sleep apnea (OSA ) in some cases [4]. The infants aged younger than 6 months even the neonates can also develop PAC [5–7]. Compared to the older children, the infants aged younger than 6 months have a superiorly placed larynx, a smaller size of pharyngeal airway as well as ventilatory control instability [7]. Furthermore, either tonsils or adenoid has not yet developed in this age [7]. So there may be differences in the comorbidities, clinical features, treatments and outcomes between the PAC with onset in the older child age and those with onset within 6-months old. In this present study, the PAC with onset within 6-months is named as early-onset PAC in infants. Until now, early-onset PAC in infants has not been given enough attention in clinic. Therefore, this present study is intended to explore the clinical features, endoscopic findings, comorbidities and outcomes of early-onset PAC in infants with the aim of extending clinician’s awareness and improving the diagnostic and therapeutic accuracy for this disease.

## Subjects and methods

### Subjects

The children of PAC with onset within 6-months old, who had been hospitalized in the Department of Pediatrics at the First Affiliated Hospital of Guangxi Medical University from October 2017 to January 2023 were included. Diagnosis of PAC needed to be confirmed by bronchoscopy. A retrospective, observational study was conducted. The present study was reviewed and approved by the Ethics Committee of the First Affiliated Hospital of Guangxi Medical University (2023-E047-01).

## Methods

### Data collection

Clinical data were collected by reviewing the medical records, including the age of onset, clinical manifestations, the performance of bronchoscopy, comorbidities, the other airway malformations rather than PAC, treatments and outcomes. Also, a follow up of the patients was performed by telephone or through outpatient visits.

### Diagnostic criteria and pattern of PAC on bronchoscopy

**Fig. 1:**
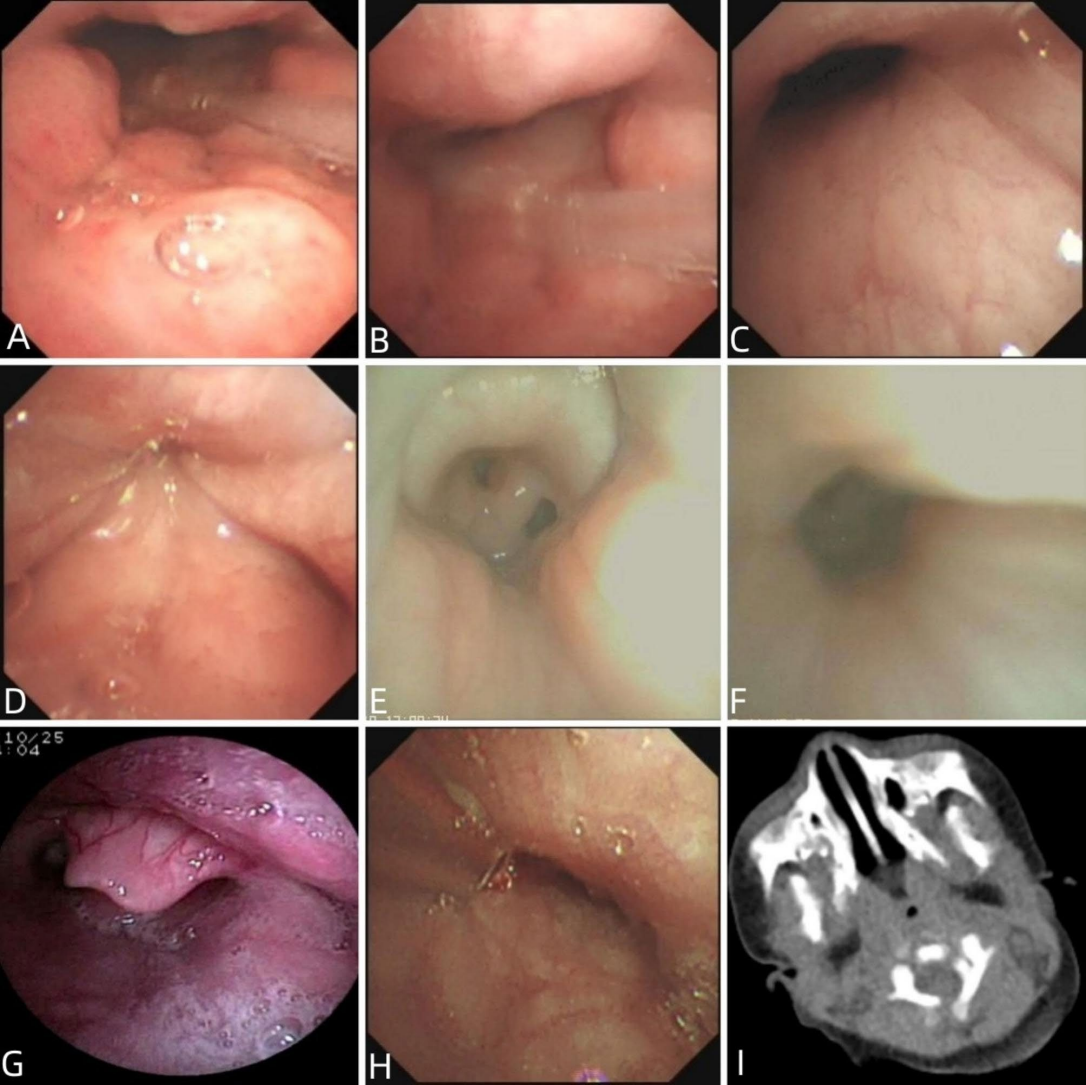
Endoscopic and CT findings of Early-onset pharyngeal airway collapse in infants **A**: antero-posterior retropalatal collapse during expiratory period; **B**: antero-posterior retropalatal collapse during inspiratory period in the same case to A; **C**: lateral retropalatal collapse during expiratory period; **D**: lateral retropalatal collapse during inspiratory period in the same case to C; **E**: lateral hypopharynx collapse during expiratory period; **F**: lateral hypopharynx collapse during inspiratory period in the same case to E; **G**: tongue base collapse; **H**: An improvement of PAL was found in the same case to C and D on bronchoscopy reviewed 1 month later; **I**: CT scan revealed pharyngeal cavity narrowing

The diagnostic criteria for PAC on bronchoscopy was that there was a more than 50% reduction of pharyngeal internal diameter, and it was more obvious during the inspiratory period [8]. According to the collapse region and the performance on bronchoscopy, 5 patterns have been identified, which included antero-posterior retropalatal collapse, lateral retropalatal collapse, lateral hypopharynx collapse, tongue base collapse and the mixed type with presence of 2 or more patterns simultaneously [2, 8–10]. (Fig. 1)

### Bronchoscopy

The children underwent a pre-operative fasting period of 2 h for liquids and 6 h for solids. Routinely, 2% lidocaine 4 ml inhalation and atropine 0.01–0.02 mg/kg subcutaneously were administered 30 min before the bronchoscopy. Firstly, the bronchoscopy was performed without use of any sedative or anesthetic drugs to assess the severity of upper airway obstruction. Then, dexmedetomidine 3–4 ug/kg intranasally and/or midazolam 0.1 mg/kg (not exceeding 0.3 mg/kg) via intravenous push were administered when needed and appropriate. Because of safety concerns, bronchoscopy was performed without use of any sedative or anesthetic drugs in the cases with severe upper airway obstruction or other conditions which were considered not to be able to tolerate the sedative or anesthetic drugs by clinicians. In the operating room, the children were placed in the supine position, and the heart rate (HR), oxygen saturation (SpO_2_) and blood presure (BP) were monitored. Bronchoscopy was performed under spontaneous breathing with unilateral nasal catheter oxygen inhalation. The flexible bronchoscope (BF-XP290; Olympus, or EB-470P; Fujinon, or QG-3320; SeeSheen) was passed through the nasal cavity to the lung. Topical anesthesia with 2% lidocaine 1ml was administrated in trachea and the main bronchus. When SpO_2_ was less than 85%, bronchoscopy was terminated, and increasing oxygen flow or pressurized mask ventilation was performed. When SpO_2_ returned to above 95%, the bronchoscopy was performed again.

### Statistical analysis

Statistical analysis was performed using SPSS 20.0 soft. Measurement data were expressed as medians (25th-75th percentile). Counting data were expressed as count or percentage. For the categorical variables, comparisons between groups were performed by the *χ*^2^ test or the Fisher exact test. A two-sided *P* value<0.05 was considered to be statistically significant.

## Results

### General information

A total of 26 cases were included, in which there were 15 male and 11 female. Five cases were preterm, the rest 21 cases were full-term. Notably, 15 cases were small for gestational age (SGA). The age of onset was neonatal period in 20 cases, 1 to 3-months old in 5 cases, 4 to 6-months old in 1 case. The median age at the time of follow up were 18.0 months (11.0, 31.0). (Table 1)

**Table 1:** Clinical data of Early-onset pharyngeal airway collapse in infants

No.	Age of onset	Pattern on bronchoscopy	CT findings	Other airway malformations	Preterm	SGA	Comorbidities	Age at follow up (months)	Prognosis	Outcome of PAC
1	neonate	LAC	not done	N	N	N	N	57	NND	improvement
2	neonate	LHC	not done	N	34^+ 1^w	Y	N	55	NND	improvement
3	neonate	APRpC and TBC	pharyngeal cavity narrowing	type1 and 3 laryngomalacia	N	N	CHARGE syndrome	18^c^	died due to airway obstruction	-
4	4 m	LAC	pharyngeal cavity narrowing	N	N	N	trisomy 21	103	SND	improvement
5	neonate	LAC and TBC	not done	type 1 laryngomalacia	N	Y	N	-	lost	-
6	neonate	APRpC	not done	type 1 laryngomalacia	N	N	N	-	lost	-
7	neonate	LAC	not done	N	N	N	duplication of 12p13.33-11.1	11 ^c^	died due to severe pneumonia	-
8	neonate	LAC and LHC	not done	tracheal stenosis (mild)	36^+ 2^w	Y	N	39	NND	improvement
9	neonate	LHC	not done	N	N	Y	1p36 deletion	31	SND	improvement
10	1 m	LAC	pharyngeal cavity narrowing	type 1 and 3 laryngomalacia	35^+ 4^w	Y	suspected MD	25	SND	improvement
11	neonate	APRpC	epiglottic cyst	epiglottic cyst	N	Y	N	28	NND	improvement
12	neonate	LAC	pharyngeal cavity narrowing	N	31^+ 4^w	Y	PCD	14 ^c^	died due to airway obstruction	-
13	neonate	APRpC	normal	N	N	N	N	13	NND	improvement
14	neonate	LAC	normal	type 1 laryngomalacia	N	Y	PRS	-	lost	-
15	neonate	LAC	not done	N	N	Y	PRS	27	SND	improvement
16	2 m	LAC	normal	subglottic hemangioma	N	N	N	22	NND	improvement
17	3 m	APRpC	normal	type 1 laryngomalacia, tracheomalacia (mild)	N	Y	N	14	NND	improvement
18	3 m	APRpC	normal	type 3 laryngomalacia	N	N	N	20	NND	improvement
19	neonate	LAC	not done	type 1 laryngomalacia	N	Y	MD^a^	16	SND	improvement
20	neonate	LAC	pharyngeal cavity narrowing, cervical hemangioma	type 1 laryngomalacia, cervical hemangioma, mild stenosis of LULB	N	N	PCD	8	NND	improvement
21	1 m	LAC	not done	N	N	N	MD^b^	11 ^c^	died due to aspiration	-
22	neonate	LAC	pharyngeal cavity narrowing	type 1 laryngomalacia, tracheomalacia (mild)	N	Y	PRS	5	SND	improvement
23	neonate	LAC	not done	type 1 and 2 laryngomalacia	N	Y	PRS	5	SND	improvement
24	neonate	LAC	not done	type 1 laryngomalacia	34^+ 6^w	Y	N	4	SND	improvement
25	neonate	LAC and TBC	not done	N	N	unknown	suspected MD	53	SND	deterioration
26	neonate	LAC	not done	N	N	Y	PCD	16	SND	improvement

^a^ The case was caused by compound heterozygous variants of *MIPEP* gene. ^b^ The case was diagnosed as Gaucher’s disease which was caused by compound heterozygous variants of *GBA* gene: *del* and *p.L483P*. ^c^ The age at death

LAC: lateral retropalatal collapse

APRpC: antero-posterior retropalatal collapse

LHC: lateral hypopharynx collapse

TBC: tongue base collapse

PCD: perinatal cerebral damage

RRS: Pierre Robin sequence

MD: metabolic encephalopathy

NND: normal neurological development

SND: survived with neurodevelopmental disorders

SGA: small for gestational age

LULB: left upper lobe bronchus

### Clinical manifestations

Noisy breathing was the most common clinical manifestation (26/26), in which 3 cases had noisy breathing only when respiratory infections occurred or in the state of agitation, whereas the rest 23 cases had noisy breathing in normal times. Suprasternal retraction was also common (18/26), in which 2 cases had suprasternal retraction when respiratory infections occurred only, whereas the rest 16 cases had suprasternal retraction in normal times. Snoring was relatively uncommon, this was found in 14 cases. Notably, hypoxic episode was found in 13 cases, in which 2 cases had hypoxic episode when asleep only, 9 cases had hypoxic episode when awake only, 2 cases had hypoxic episode when both asleep and awake. The triggers of hypoxic episode which occurred when awake included agitation in 6 cases, secretion obstruction in 3 cases and sucking in 1 case.

### Endoscopic findings

Among the 26 cases, 5 cases were classified as antero-posterior retropalatal collapse, 15 cases were classified as lateral retropalatal collapse, 2 cases were classified as lateral hypopharynx collapse, 4 cases were classified as the mixed pattern. In the cases with mixed pattern, there were antero-posterior retropalatal and tongue base collapse in 1 case, lateral retropalatal and tongue base collapse in 2 cases, lateral retropalatal and lateral hypopharynx collapse in 1 case. In summary, collapse at the retropalatal level was most common, this was found in 24 cases. (Fig. 1; Table 1)

### CT findings

Total 12 cases underwent the pharyngolaryngeal CT examination, which revealed abnormal findings in 7 cases. Of these cases with abnormal CT findings, 5 cases presented with pharyngeal cavity narrowing, 1 case presented with pharyngeal cavity narrowing as well as cervical hemangioma, 1 case presented with epiglottic cyst. (Fig. 1)

### Other airway malformations rather than PAC

Fifteen cases were accompanied with the other airway malformations rather than PAC. Congenital laryngomalacia (CL) was most common, which was found in 12 cases. (Table 1)

### Comorbidities

Fifteen cases had comorbidities of cerebral impairment and/or craniofacial abnormalities, in which 6 cases were the congenital diseases due to the gene variant, 2 cases were perinatal cerebral damage, 4 cases were Pierre Robin Sequence (PRS), 3 cases were suspected metabolic encephalopathy. (Table 1)

### Treatments

The majority of the cases (21/26) needed supplementary oxygen therapy or respiratory support therapy, 1 case needed tracheostomy. Four cases with CL received supraglottoplasty through bronchoscopy, an improvement of dyspnea was found in all the 4 cases. Notably, an improvement of PAC was found in 1 case on bronchoscopy reviewed 1 month later. (Fig. 1)

### Prognosis and outcomes of PAC

According to the presence of comorbidities of cerebral impairment or craniofacial abnormalities or not, the cases were divided into the group with comorbidities (15 cases) and that without comorbidities (11 cases). In the group with comorbidities, 1 case was lost to follow up, 4 cases died, and 10 cases survived, in which 9 of the survived cases had neurodevelopmental disorders. The cause of death included severe airway obstruction in 2 cases, severe pneumonia in 1 case and aspiration in 1 case. In the group without comorbidities, 2 cases were lost to follow up, 9 cases survived, in which only 1 case of the survived cases had mild neurodevelopmental disorders, and the rest 8 survived cases had normal neurological development. The incidence of poor prognosis including death and neurodevelopmental disorders was significantly higher in the group with comorbidities than that without comorbidities (95.9% vs. 11.1%, *P<0.01*). It is worth noting that an symptomatic improvement of PAC was found in the majority of the survived cases (18/19) with age. (Table 1)

## Discussion

Pharyngeal airway performs as a transportation hub to connect the nasal cavity, oral cavity and the glottis. Normally, the support of the pharyngeal airway wall is strong enough to prevent the pharyngeal airway from significantly collapsing. When the support of the pharyngeal airway wall is weakened, the pharyngeal airway may collapse significantly. It can result in airflow limitation, especially during the inspiratory period [11]. Until now, the PAC in older children has been well known to clinicians, whereas the PAC with onset within 6-months old which was named as early-onset PAC in infants in this present study has not been given enough attention in clinic. As the characteristics of respiratory anatomy and physiology are different in the infants of less than 6-months old and older children, there may be differences in the comorbidities, clinical features, treatments and outcomes between the PAC with onset within 6-months old and those with onset in the older child age.

Usually, PAC is diagnosed objectively as well as preferably by polysomnography [5]. Regrettably, we haven’t carried out polysomnography in the children younger than 2 years old in our hospital. Also, it seemed to be not applicable for the cases of early-onset PAC in infants, as some cases were too severe to tolerate this examination, and some cases couldn’t receive this examination due to the respiratory support therapy using ventilation tube. In addition to polysomnography, endoscopy is useful for diagnosis and evaluation of PAC. In adult, the endoscopic pattern of PAC has been well explored, and a fundamental consensus has been reached. According to the collapse region and the performance on bronchoscopy, total 6 patterns have been identified in adult, which included antero-posterior retropalatal collapse, lateral retropalatal collapse, enlargement of the lymphoid tissue (tonsils and adenoid), lateral hypopharynx collapse, tongue base collapse and epiglottic collapse [2, 8–10]. In this present study, the endoscopic pattern of early-onset PAC in infants was referred to those in adult. In children, the epiglottic collapse due to the congenital factors has been grouped as type 3 laryngomalacia [12], so the epiglottic collapse was not included in the endoscopic pattern of early-onset PAC in infants in this present study. As mentioned above, PAC is mainly secondary to enlargement of the lymphoid tissue (tonsils and adenoid) in older children. Whereas, either tonsils or adenoid has not yet developed in the infants of less than 6-months old [7], thus it is very unlikely that enlargement of the lymphoid tissue (tonsils and adenoid) can result in PAC in the infants of less than 6-months old. For this reason, the enlargement of the lymphoid tissue (tonsils and adenoid) was not included in the endoscopic classification of early-onset PAC in infants here. In this present study, it was found that collapse at the retropalatal level was most common, whereas lateral hypopharynx collapse was relatively uncommon. Of the cases with collapse at the retropalatal level, lateral retropalatal collapse was more common than antero-posterior retropalatal collapse. Compared to bronchoscopy, CT scan can’t dynamically monitor change of pharyngeal airway, Due to this reason, CT scan is not the traditionally preferred choice for PAC. While, in this present study, CT scan could reveal pharyngeal cavity narrowing in half of the cases and help to identify other airway malformations rather than PAC. In view of this, CT scan may be is a valuable addition to a diagnostic evaluation of PAC with onset within 6-months old.

Comorbidities should be investigated in the patients with PAC. Unlike PAC in the older children who required attention to the enlargement of the lymphoid tissue (tonsils and adenoid), the early-onset PAC in infants required more attention to the congenital craniofacial anomalies, such as RPS, syndromic craniosynostosis [6, 7]. Also, cerebral impairment due to the congenital neurodevelopmental abnormalities, cerebral ischemic/hypoxic injury or intracerebral hemorrhage can lead to neural dysregulation of upper airway muscle activity and result in the PAC [13]. It is worth noting that some congenital diseases due to the gene variant, such as CHARGE syndrome [14], 1p36 deletion syndrome [15] can develop both craniofacial abnormalities and cerebral impairment. In this case, it is more likely to develop PAC. In this present study, more than half of the cases had the comorbidities, which was in accordance with the literature. In addition to the factors mentioned above, both preterm and gastro-oesophageal reflux are considered to contribute to the pathogenesis of the early-onset PAC in infants [7]. In this present study, preterm was presumed to be the single risk factor for the PAC in 3 cases, whereas no case of gastro‐oesophageal reflux was found which might have been ignored. Furthermore, SGA was relatively common in this present study. It demonstrated that intrauterine growth retardation was related to the pathogenesis of early-onset PAC in infants, which deserved further study.

In the older children with PAC, it usually presents as snoring [16]. The limited clinical data have showed that the clinical features of early-onset PAC in infants were different from the PAC with onset in the older child age [7]. In the present study, the most common clinical manifestation of early-onset PAC in infants was noisy breathing, whereas snoring was relatively uncommon. Usually, PAC with onset in older child age dose not manifest as airway obstruction when awake. Different from this, airway obstruction when awake was relatively common in the early-onset PAC in infants. In this study, suprasternal retraction could be found in the more than half of the cases. Remarkably, half of the cases had hypoxic episode in this present study. In the PAC with onset in older child age, hypoxic episode usually occurs when asleep, which is related to the OSA [16]. In this present study, hypoxic episode were more common in the state of agitation in the early-onset PAC in infants, rather than asleep. The difference of comorbidities as well as characteristics of respiratory anatomy and physiology might contribute to the remarkable difference in the clinical manifestations [7].

In this present study, CL was found to be common in the early-onset PAC in infants. The pathogenesis of CL is predominantly related to the supraglottic abnormal anatomical structures including prolapse of the mucosa or soften cartilage and the neural dysregulation of upper airway muscle activity [17]. Mechanistically, there are similarities between the early-onset PAC in infants and CL. Also, early-onset PAC in infants can be accompanied with lower respiratory malformation [6]. In this present study, 5 cases were found to be accompanied with lower respiratory malformation. Compared to the nasal endoscopy, the bronchoscopy can reveal both the upper and lower respiratory tract. In view of this, bronchoscopy may be preferred to be the choice in the case of early-onset PAC of infant to avoid missing diagnosis of the accompanied lower respiratory malformation.

The treatment strategy for early-onset PAC in infants should be individualized according to the severity of diseases, comorbidities and the wishes of the patient’s family [6, 7]. The treatment methods include treating of the comorbidities, changing position, oxygen therapy, noninvasive ventilation (CPAP or BiPAP) as well as nasopharyngeal airway intubation, and invasive ventilation or tracheostomy may be necessary in some cases [6, 7]. Sometimes, mandibular distraction or mandibular distraction osteogenesis are required in the cases caused by craniofacial abnormalities [7]. In this present study, 4 cases with CL had received supraglottoplasty. Remarkably, there was a clinical improvement of dyspnea in all the 4 cases. Also, an improvement of PAC was found in 1 case on bronchoscopy reviewed 1 month later. It demonstrated that treatment of co-morbid disorders of CL actively might contribute to the relief of PAC.

Until now, there have been limited studies on the prognosis of early-onset PAC in infants. In this present study, the majority of the survived cases presented a symptomatic improvement of PAC with age. The reasons for this phenomenon might be related to the rapid growth of craniofacial skeleton and neurological maturation [6, 7]. The growth of craniofacial skeleton may enlarge the internal diameter of pharyngeal airway, and the neurological maturation may improve the neural regulation of upper airway muscle activity, both of which can contribute to the relief of PAC [6, 7]. However, early-onset PAC in infants is related to sudden infant death syndrome, especially in the cases with comorbidities of cerebral impairment and/or craniofacial abnormalities [7, 18], which need to attract clinician attention. In addition, neurodevelopmental disorders may be caused by the potential comorbidities or the recurrent hypoxic episode. A further follow-up of the long-term prognosis of neurological development was necessary in the early-onset PAC in infants [6, 7]. In this present study, the majority of the cases with cerebral impairment and/or craniofacial abnormalities had neurodevelopmental disorders, whereas the majority of the cases without comorbidities mentioned above had normal neurological development. It demonstrated that the long-term prognosis of neurological development was closely related to the comorbidities.

Nevertheless, there were some limitations in this present study. As this study was a single center, small sample size and retrospective clinical study, which might result in selection and recall bias. In the future, a multiple centers, large sample size and prospective clinical study should be performed.

## Conclusions

Early-onset PAC in infants has its own unique and distinctive features of clinical manifestations, endoscopic findings, treatments and outcomes. It usually exhibits varying degrees of relief with age, whereas the cases with comorbidities had a poor prognosis.

## Data Availability

All data generated or analyzed during this study are included in this published article.

## Abbreviations

PAC: Pharyngeal airway collapse
OSA: Obstructive sleep apnea
CL: Congenital laryngomalacia
PRS: Pierre Robin Sequence
LAC: Lateral retropalatal collapse
APRpC: Antero-posterior retropalatal collapse
LHC: Lateral hypopharynx collapse
TBC: Tongue base collapse
PCD: Perinatal cerebral damage
MD: Metabolic encephalopathy
NND: Normal neurological development
SND: Survived with neurodevelopmental disorders
SGA: Small for gestational age
LULB: Left upper lobe bronchus
